# Effects of substance use disorder on treatment process and outcome in a ten-session psychiatric treatment for borderline personality disorder

**DOI:** 10.1186/s13011-018-0145-6

**Published:** 2018-02-26

**Authors:** Louise Penzenstadler, Stéphane Kolly, Stéphane Rothen, Yasser Khazaal, Ueli Kramer

**Affiliations:** 1 Geneva University Hospitals, Service d’addictologie HUG, Rue de Grand-Pré 70c, 1202 Genève, Switzerland; 2 Department of Psychiatry, University Hospital of Lausanne, University of Lausanne, Lausanne, Switzerland; 3 Department of Psychiatry, Faculty of medicine, Geneva University, Geneva, Switzerland; 4 Research Center, Montreal University Institute of Mental Health, Montréal, Canada; 5 Department of Psychology, University of Windsor, Windsor, Canada; 6 Research Center for Statistics, Geneva School of Economics and Management, University of Geneva, Geneva, Switzerland

## Abstract

**Background:**

Dual diagnosis is common in Borderline Personality Disorder (BPD), one of the most common being Substance Use Disorder (SUD). Previous studies have shown that general psychiatric management (GPM) was effective in reducing borderline symptoms.

In the present study, we tested whether the short GPM was as effective in the BPD + SUD as in the BPD group.

**Methods:**

We analysed a group of 99 patients presenting a BPD. 51 of these patients presented a SUD. The BPD group and the BPD + SUD group received a manual-based short variant of the GPM treatment. Previous studies have shown that a 10-session version of GPM was effective in reducing borderline symptoms at the end of the treatment (Psychother Psychosom 83:176–86, 2014).

**Results:**

We found no significant difference in the reduction of general symptoms, which diminished in both groups. The specific borderline symptoms were also reduced in both groups, but there was a slightly higher reduction of the borderline symptoms in the SUD group. The therapeutic alliance progressed positively in all groups. Moreover, the alliance increased more over time in the SUD group.

**Conclusion:**

The short variant of GPM seems to be effective in BPD treatment independently from the presence of SUD. Therefore, this treatment could be an effective entry-level treatment for patients with dual diagnosis as well as patients with BPD only. Further studies are needed to confirm efficacy and long-term outcome.

**Trial registration:**

The trial was registered at ClinicalTrial.gov (identifier NCT01896024).

## Background

Borderline personality disorder (BPD) is a common mental disorder. It is associated with high suicide rates and severe functional impairment [1, 2]. Patients suffering from BPD have high rates of co-morbid mental disorders, amongst them substance use disorders (SUD) are very common. According to a systematic review, about 38–57% of BPD patients also have a SUD diagnosis [3]. On the other hand, between 5 and 32% of people with SUD are estimated to meet the criteria for BPD [4, 5].

Some studies reported more severe SUD profile for patients with BPD [6]. Studies show inconsistent findings concerning a possible aggravation of BPD symptoms by SUD [7]. Overall people with this co-morbid condition seem to have higher rates of depression and lower levels of functioning [8, 9].

The higher levels of psychosocial impairment, more severe psychopathology and the increased rates of suicidal behavior of the co-morbid BPD with SUD population presents a considerable challenge for mental health services [10–12].

BPD is a chronic illness for which several evidence based treatment models have been developed. Most of these are long term very specialized and costly treatments which limits treatment accessibility [13–15].

Only few randomized trials with small study populations assessed treatment programs in co-morbid patients [7, 16].

Treatment retention is an issue for some patients presenting a SUD, some analysis show earlier drop-out [17, 18] and some do not [19, 20]. Especially women drop out more likely when presenting higher levels of psychiatric symptoms [21]. In order to offer full treatment cycles to difficult to engage patients, shorter treatment packages could be helpful.

As for other psychiatric disorders short-term treatment models [22, 23] allow more patients to benefit from therapy and to make better use of limited resources. An effective treatment for BPD is the general psychiatric management (GPM) developed by Gunderson [24]. Generalist mental health clinicians can deliver this treatment after a short introductory course. It focuses on psycho-education, informed management of medication and co-morbid disorders. The aim is to improve the patient’s functionality and quality of life. Various studies reported that the model or versions of it are effective for BPD [25, 26] but so far no studies have specifically examined its effect when treating a BPD population with substance use disorder in a short-term time frame. This is the objective of the present study.

Short-term treatments are interesting for patients with SUD as these patients are generally more difficult to engage in long-term psychotherapy treatments due to the chronic and relapsing nature of SUD [27, 28].

Paris [29] and Chanen and Thompson [30] suggest a stepped-care model in which different evidence-based treatment models for BPD by stepwise progression for different clinical phases [31]. According to this model, short-term treatment are useful and cost-effective first steps strategies to help BPD patients manage their symptoms in order to improve their functionality [31]. When they are more organized and can enter more in depth psychological treatment, they can be referred to specialists for psychotherapy. This approach is of high interest for patients with SUD as the mental health practitioner for SUD can easily apply psychiatric interventions without having to address the patient to a BPD specialized therapist. Often patients with SUD are not stable enough to regularly attend fixed group meetings like for DBT and are therefore often excluded from such programs. A study by Black shows that patients with higher baseline severity showed greater improvement after short-term treatment [32]. As mentioned above the cost-effectiveness of GPM is an important advantage over long-term treatments by specialized practitioners. The shorter GPM consists of weekly sessions which requires less intensive training. The ability of this model to address more patients with a proven effective treatment can therefore reduce direct and indirect costs caused by BPD [33]. Direct costs are due to hospital admissions, emergency room visits and primary care visits and indirect costs are those caused by other medical and social problems related to lower income, marriages and child-rearing difficulties [34, 35].

In spite of such promising rationale for the use of GPM with people with co-morbid BPD and SUD, we expect a negative impact of the co-morbidity on the GPM treatment outcomes given the increased severity associated with this co-morbidity in comparison to BPD alone [10–12]. The fact that these patients often show a higher symptom level [36] and more psychiatric comorbidities is usually associated with the conclusion that they would show worse outcome in any treatment. The higher risk of treatment drop-out due to active substance use and a possibly less strong therapeutic alliance could be a reason for this negative outcome.

On the other hand, treating patients with GPM could actually make patients with BPD and SUD feel better understood as this treatment focuses more largely on their problems and does not focus solely on SUD and associated symptoms. This might help therapists adapt better to the patients’ needs and could actually improve the therapeutic alliance. Also, SUD does not always negatively influence therapeutic alliance [37]. The.

To our knowledge no short-term program has been studied for patients with co-morbid BPD and SUD.

### Aims of the study

The aim of the study at hand is to compare the impact of a 10-session version of a GPM treatment [24, 38] on patients with BPD and patients with BPD and a co-morbid SUD concerning treatment process and outcome.

### Hypotheses

1. We assume that the presence of a co-morbid substance use disorder diminishes the symptom change found over ten sessions of psychiatric treatment.
2. We assume that the presence of a co-morbid substance use disorder diminishes the quality of the therapeutic alliance over the course of the first ten sessions of therapy.

## Methods

### Design

In the present study we examined the role of a co-morbid substance use disorder on the process and outcome of a 10-session version of an APA-informed psychiatric treatment (“good psychiatric management”; GPM [24]) for BPD.

The present study is a secondary analysis of two randomized controlled studies, both originally examining the identical research question and using identical procedures, which justifies their combination for the present study [39, 40]. Together the two studies randomized a total of 114 (29 plus 85, respectively) patients with BPD to two conditions based on a 10-session treatment for BPD: a short version of the GPM in the first condition, and the same treatment augmented with the motive-oriented therapeutic relationship (MOTR) [41]. Adherence to GPM model was excellent for both conditions [42]. Kramer, Kolly et al. [40] found significant symptom reduction for both conditions on all measures, between condition analyses revealed between-group effects varying between d = 0.06 for specific borderline symptom and d = 0.64 for general symptom outcome reduction. Adding MOTR to GPM showed a greater reduction of general problems. However no additional borderline symptom reduction was found in the MOTR group.

The reporting and discussion of missing data in this study, along with analyses of treatment drop-out, are provided in the original publications [39, 40].

In these former studies the impact of SUD was not specifically examined. In the present research we examine the impact of SUD on the treatment outcome.

In consideration of the lack of differences in outcome measures for GPM and GPM + MOTR in the previous studies we consider it as acceptable to combine the two groups in order to improve our sample size to maximize power. Both groups received the same GPM 10-sessions treatment.

In order to assess differences in alliance and outcomes between patients with BPD and patient with comorbid SUD, we differentiated between (a) patients with a comorbid alcohol use disorder (AUD), (b) patients with other SUD (OSUD; all drugs included), (c) patients with both AUD and OSUD, and (d) patients with no SUD co-morbidity.

### Patients

A total of *N* = 114 patients were considered for analysis in the present sample. Because of missing diagnostic information (4 in the pilot study and 11 in the main study), 99 patients were finally included in the present analysis. All patients were treated at a French speaking outpatient university clinic. All patients presented a DSM-IV diagnosis of BPD. The ethnic composition represented the consulting psychiatric population in public European clinical services (85% Caucasian). Diagnoses were established by trained clinicians using the Structured Clinical Interview for DSM-IV Axis II Disorders [43], along with the Mini Neuropsychiatric Interview for co-morbid psychiatric disorders [44]. The reliability of the DSM-IV axis II diagnosis was satisfactory; κ = 0.81. Reliability was analyzed with independent ratings of video-taped SCID-II diagnostic interviews on a randomly chosen 10% of all patients included [40]. The addiction diagnosis was based on chart review using DSM-IV criteria. The diagnoses were made by the psychiatrist treating the patient. SUD diagnostic accuracy was routinely supervised by a senior psychiatrist. In total, *n = 20* (20%) presented a co-morbid alcohol use disorder, *n = 10 (10%)* presented another SUD, *n = 21 (21%)* presented alcohol and another SUD concomitantly, the remaining patients, *n = 48 (48%)* are without any co-morbid SUD (see Table 1). The drop-out rate was 25% for patients without SUD, 40% for patients with alcohol use disorder, 0% for patients with another SUD and 24% for patients with alcohol and comorbid SUD.

**Table 1:** Baseline characteristics

Variables	Condition						*p* value^*^	
	None *n* = 48(%)	Alcohol *n* = 20 (%)	Other SUD *n* = 10(%)	Both *n* = 21 (%)				
female	16 (33)	9 (45)	1 (10)	5 (24)			0.234	
Marital status							0.396	
Never married	25 (52)	8 (40)	3 (30)	14 (67)				
Married	13 (27)	9 (45)	4 (40)	4 (19)				
Separated, divorced	10 (21)	3 (15)	3 (30)	3 (14)				
Employment							0.450	
Unemployed	35 (73)	16 (80)	7 (70)	17 (81)				
Protected activity	2 (4)	2 (10)	1 (10)	2 (10)				
Part-time	2 (4)	1 (5)	2 (20)	1 (5)				
Full-time	8 (17)	1 (5)	0 (0)	1 (5)				
Treatment status							0.116	
completers	36 (75)	12 (60)	10 (100)	16 (76)				
drop-out	12 (25)	8 (40)	0 (0)	5 (24)				
					Test value	df	*p* value	Post-Hoc
Age^a^, years								
mean	32.6	36.8	28.1	29.0	2.562	(3,95)	0.059	
s.d.	9.7	11.3	7.6	11.8				
Current DSM-IV diagnoses^b^								
number of Axis I diagnoses (mean)	1.40	0.75	1.40	0.95	13.207	3	**0.004**	None vs OH^c^
(s.d.)	0.77	0.72	0.97	1.16				
Presence of Axis II diagnoses^*^								
n	22	8	0	5			0.016	None vs Other SUD^d^
%	46.8%	40.0%	0.0%	23.8%				
Education^a^, years								
mean	11.74	10.65	10.70	11.14	2.235	(3,94)	0.089	
s.d.	1.84	1.39	1.95	2.01				
OQ-45^a^								
mean	92.19	92.95	102.00	101.00	1.101	(3,95)	0.390	
s.d.	24.15	18.71	32.38	22.08				
BSL^a^								
mean	1.80	1.80	1.65	1.94	0.132	(3,58)	0.941	
s.d.	0.91	1.10	1.13	0.79				
GAF^a^								
mean	63.02	61.25	62	59.095	0.955	(3,95)	0.417	
s.d.	8.55	9.44	9.19	9.42				
Number of BPD symptoms^a^								
mean	6.35	6.85	6.80	7.00	1.360	(3,95)	0.260	
s.d.	1.42	1.35	1.40	1.30				
WAI^a^								
mean	57.22	52.77	57.36	60.96	0.974	(3,78)	0.409	
s.d.	11.70	18.00	12.49	15.09				

Values are expressed as numbers (with percentages in parentheses) or as means

*OQ-45* Outcome Questionnaire – 45.2, *BSL* Borderline Symptom List, *GAF* Global Assessment of Functioning, *BPD* Borderline Personality Disorder, *WAI* Working Alliance Inventory

^*^Fisher’s exact test

^a^One-way ANOVA

^b^Kruskal-Wallis rank sum test

^c^Significant results from post-hoc test: Mann–Whitney-Wilcoxon test, significant threshold set at 0.05/6 = 0.0083

^d^Significant results from post-hoc test: Fisher’s exact test, significant threshold set at 0.05/6 = 0.0083

Significant *p*-values are in bold

### Therapists

In total, *N* = 24 therapists (13 of them treated 3 patients or less, and 11 more than 3 patients) conducted the treatments of the patients in this study. All the therapists were trained in the clinical procedures related to the psychiatric management of BPD in 10 sessions before the study began. The therapists were residents in psychiatry for an overall mean of 2.5 years (at least one year).

### Treatment

A short-term treatment program held in 10 sessions based on the principles of the good psychiatric management for BPD (GPM) was used in this study. The therapists conducted the treatment according to a manual focusing on GPM treatment principles: [1] establishment of a reliable psychiatric diagnoses, including co-morbidities and other problem areas, and communication of this information to the patient; [2] synthesis of the psychiatric anamnesis; [3] identification of the treatment focus; [4] definition of short-term objectives and enhancement of treatment motivation; [5] working on treatment-interfering problems, and [6] formulation of attachment-based core conflictual themes. In general, the patients received one session per week; if necessary, short-term inpatient treatment and psychopharmacological treatment was added.

### Instruments

*Outcome Questionnaire – 45.2 (OQ-45)*, is a self-report questionnaire which assesses results from psychotherapy using 45 items. It includes a global score and three sub-scores: symptomatic level, interpersonal relationships and social role [45]. These items are rated on a Likert-scale ranging between 1 (never) and 4 (always). This questionnaire is widely used and has been translated and validated in French [46]. This scale was given measured at intake and discharge. Cronbach’s alpha at intake of the present sample was .91.

*Borderline Symptom List (BSL-23)*, [47], which is also a self-report questionnaire. This measures specific borderline symptomatology using 23 items. The specific items are measured using a Likert-type scale ranging from 0 (absent) to 4 (clearly present) and an overall mean score is retained. It is a shortened version of the BSL-95, which reportedly has very good psychometric properties. Similar results were found for the short version. The present study used the French translation approved by the authors. Cronbach’s alpha for the current sample was .95.

*Working Alliance Inventory – short form (WAI-short version)*, which is a self-report questionnaire, of 12 items assessing the different dimensions of therapeutic alliance, the bond between patient and therapist and therapy collaboration agreement (task and goals) [48]. The items are measured on a Likert-type scale ranging from 1 (never) to 7 (always); an overall sum score is computed [49]. The questionnaire measuring the therapeutic alliance was given to the patient after each session.

### Procedure

The responsible Ethic Committee cleared the study. After an intake interview, the patients meeting inclusion criteria were met by a program related researcher who explained the study to them. After written informed consent, these patients then received 10 sessions of GPM according to the manual. During the treatment, they answered the OQ-45 and the BSL-23 at intake and discharge.

### Statistical analyses

Characteristics at baseline are presented within four groups of patients: BPD with no substance or alcohol use disorder, BPD with alcohol use disorder only, BPD with substance use disorder only and BPD with both substance and alcohol use disorder. In order to compare these four groups, Fisher’s exact test (instead of Chi-square due to the small sample size) or ANOVA or Kruskal-Wallis rank sum test were performed.

In order to test hypothesis 1) stating that the SUD lessened the symptom decrease over the course of treatment, we first conducted two paired-sample t-tests on the score of OQ-45 and BSL-23, the grouping variable being presence of SUD. As a measure of effect size, which is a magnitude of the difference between groups or time points, Cohen’s d were also computed [50]. According to Cohen [50], d smaller than 0.20 is considered as a small effect, between 0.20 and 0.50 as a medium effect size and greater than .50 as large effect size. And second, we conducted two parallel Linear Mixed-effects Models [51] with the score of OQ-45 and BSL-23 as the dependent variables, SUD (None vs. Any SUD) and Time (intake vs. discharge) as the independent variables as well as age, gender and therapeutic condition (BPM vs. BPM + MOTR) as covariates. This type of model is suitable for correlated measurements [52] as it accounts for the lack of independence of the observations due to the fact subjects are measured more than once.

In order to test hypothesis 2) stating that the SUD lessened the alliance level and progression over the first 10 sessions of treatment, we also ran a Linear Mixed-effects Models with the alliance measured by the WAI as the outcome, SUD (None vs. Any SUD) and Session number as the independent variables as well as age, gender and therapeutic condition (BPM vs. BPM + MOTR) as covariates. At least 2 measurement occasions are required for a patient to be included in the analyses.

Statistical analyses were performed using R 3.3.0 [53]. Regarding the Linear mixed-effects models, the ‘nlme’ R package [54] has been used [51, 52].

## Results

### Preliminary analyses (see Table 1)

*N* = 99 patients were included in the present analysis. In total, *n* = 68 (69%) were female. The average age was 32.2 years (s.d. = 10.6, media*n* = 30, range:19–55), *n* = 50 (50%) had never been married, *n* = 30 (30%) were married and *n* = 46 (46%) were separated or divorced. The employment rate was very low. *n* = 75 (74%) were unemployed, *n* = 7 (7%) were working in a protected activity, *n* = 6 (6%) were had a part-time job and *n* = 10 (10%) were working full-time. *n* = 74 (73%) completed the treatment, in the alcohol use disorder (AUD) group the number of completers was slightly lower than in the other groups, *n* = 12 (60%). Further details of each sample can be found in Table 1.

#### Characteristics at baseline

No between-group effects reached statistical significance for all variables at baseline except for number of axis I diagnoses. Contrary to expectation, post-hoc test revealed that patients without substance use had significantly more axis I diagnosis (cf. Table 1). Also for the outcome variables, no between group differences at intake (OQ-45, BSL and WAI) were statically significant.

Since no statistical differences were found between those four groups, except for axis 1 (difference None vs. Other SUD) and axis 2 (difference None vs. Alcohol) diagnoses, Alcohol and substance use disorder have been merged in order to create two groups only: BPD without any SUD, *N =* 48 and BPD with SUD, *N* = 51.

### First hypothesis: Effect of SUD on symptoms over the course of treatment

#### OQ-45 – Questionnaire

The OQ-45 was measured at intake and discharge. It assesses results from psychotherapy including symptomatic level, interpersonal relationships and social role. Overall, OQ-45 improved between intake and discharge (mean and s.d. at intake = 95.2±23.7, mean and s.d. at discharge = 81.9±24.0, mean difference = − 13.23, paired *t*-test: t = − 6.35, df = 98, *p* < 0.001;d = 0.56). When comparing Any SUD with no SUD/None there was no significant difference between OQ-45 at intake and at discharge (mean difference and sd for no SUD = − 14.1±22.6, mean difference and sd for Any SUD = − 12.6±19.3, independent *t*-test on mean difference by groups, t = − 0.369, df = 97, *p*-value = 0.71;d = 0.07).

When controlling for potential confounders (cf. Table 2), results regarding Any SUD vs no SUD/None, as well as evolution over time are the same. Interestingly, the GPM + MOTR condition is significantly associated with an improvement of symptoms over time.

**Table 2:** Effects of SUD on symptoms over the course of treatment, results from the linear mixed-effects models

	OQ-45			BSL		
	β	Std. Error	*p*-value	β	Std. Error	*p*-value
Age	0.54	0.20	**0.009** ^1^	0.03	0.01	**0.023** ^3^
Gender (Male vs. Female)	−2.67	4.59	0.563^1^	− 0.13	0.24	0.579^3^
Any SUD vs. None	6.03	7.41	0.418^1^	0.46	0.38	0.236^3^
GPM + MOTR vs. GPM	15.94	7.45	**0.035** ^1^	0.20	0.39	0.605^3^
Time (Intake or Discharge)	−7.76	3.60	**0.033** ^2^	0.01	0.19	0.967^4^
Any SUD x Time	0.46	4.07	0.910^2^	− 0.44	0.21	**0.037** ^4^
GPM + MOTR x Time	− 12.22	4.07	**0.003** ^2^	− 0.12	0.21	0.557^4^

*SUD* Substance Use Disorder, *GPM* General Psychiatric Management, *MOTR* Motive-oriented therapeutic relationship, *OQ-45* Outcome Questionnaire – 45.2, *BSL* Borderline Symptom List

Significant *p*-values are in bold

^1^T-test with 94 degrees of freedom

^2^T-test with 96 degrees of freedom

^3^T-test with 57 degrees of freedom

^4^T-test with 58 degrees of freedom

#### BSL-23 – Questionnaire

The BSL-23 which measures the number of specific borderline symptoms was given at intake and discharge. Regarding the number of specific borderline symptoms (BSL-23), only 61 patients were included in the analysis since this questionnaire was added later in the study. There was an overall improvement (mean and s.d. at intake = 1.8±0.9, mean and s.d. at discharge = 1.5±1.0, mean difference = − 0.26, paired *t*-test: t = − 2.544, df = 60, *p* < 0.014; d = 0.28) with a better improvement within the Any SUD group (mean difference and sd for no SUD = − 0.07±0.81, mean difference and sd for Any SUD = − 0.49±0.78, independent *t*-test on mean difference by groups, t = 2.074, df = 59, *p*-value = 0.04;d = 0.53).

**Fig. 1:**
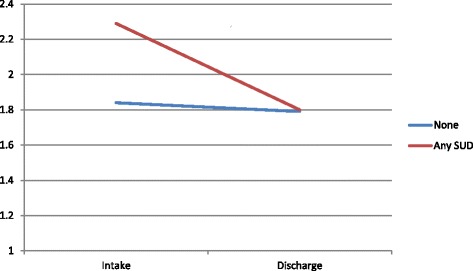
Adjusted change of the BSL from the linear mixed-effects models

When controlling for potential confounders (cf. Table 2), there is a significant interaction between Time and SUD co-morbidity, in the sense that the improvement is better within the Any SUD group (cf. Fig. 1). In this case, GPM + MOTR condition was not significantly associated with a change of BSL-score over time.

### Second hypothesis: Effects of SUD on the therapeutic alliance

The analyses on the possible impact of SUD on the therapeutic alliance were done on a sub-sample of *n* = 82 patients, due to *n* = 17 patients with missing alliance data. For the computation of the progression, *n* = 75 patients were included. In order to be included in this latter analysis, the patients needed at least two assessment points of the therapeutic alliance (*n* = 7 patients only had one data point for the therapeutic alliance).

When taking into account the intra-subject variability of the alliance, substantiated as the session-by-session alliance progression over the course of treatment (cf. Table 3), we found the following picture. The progression of the therapeutic alliance was positively significant in the analyses (regression coefficient: cf. Table 3). Moreover, the therapeutic alliance increases more over time in the Any SUD category comparing to the None category (regression coefficient: cf. Table 3). There is no statistically significant difference between GPM + MOTR and GPM condition (regression coefficient: cf. Table 3).

**Table 3:** Effects of SUD on the therapeutic alliance, results from the linear mixed-effects models

	WAI				
	β	Std. Error	d.f.^a^	*t*-value	*p*-value
Age	−0.02	0.15	70	−0.16	0.871
Gender (Male vs. Female)	−1.97	3.43	70	−0.57	0.568
Any SUD vs. None	−3.43	3.21	70	−1.01	0.288
GPM + MOTR vs. GPM	−3.03	3.28	70	−0.92	0.360
Session	0.52	0.25	410	2.11	**0.036**
AnySUD x Session	0.71	0.28	410	2.55	**0.011**
GPM + MOTR x Session	0.08	0.28	410	0.27	0.786

*SUD* Substance Use Disorder, *GPM* General Psychiatric Management, *MOTR* Motive-oriented therapeutic relationship, *WAI* Working Alliance Inventory

Significant *p*-values are in bold

^a^degrees of freedom

## Discussion

As mentioned previously SUD and BPD are highly co-morbid and the present study aims to compare the impact of a GPM treatment [24, 38] on patients with BPD and patients with BPD and a co-morbid SUD.

In the initial studies by Kramer and Kolly [39, 40] the effect of adding motive-orientated therapeutic relationship (MOTR) to GPM was analyzed regarding symptom reduction and therapeutic alliance. Both groups showed a significant reduction of general symptoms (OQ-45) and specific borderline symptoms (BSL-23). The individualizing approach in the MOTR group did not have an additional effect on the reduction of specific borderline symptoms but did have an effect on the decrease of the general and interpersonal problems. As GPM is already aimed at specifically reducing these, we did not expect much room for improvement on the borderline symptoms. The therapists in the MOTR group rated a higher alliance progression. It is possible that the treatment period was too short for the patients to percept this improved alliance due to its focus on patient’s motives.

In our secondary analysis we examined the effect of alcohol and other substance use disorders on the therapy outcome after 10 sessions of GPM. As explained earlier consistent results were found when differentiating in 4 groups (AUD, Other SUD, AUD and SUD, None). In order to increase power, we only report the results from the Any SUD and None groups.

We hypothesized that SUD would diminish the symptom change found over 10 sessions of psychiatric treatment. After 10 sessions, we observed a significant reduction of borderline symptoms according to the BSL-23 in both groups. However, in contradiction with our hypotheses, in the group of patients presenting a SUD the borderline symptom reduction was more important than in the group without this co-morbid condition (See Fig. 1), moderate effect size on BSL-23.

This is in line with the observation made by Black et al. [32]. Patients with higher baseline severity scores improve more than those with lower scores at baseline after a short-term intervention. Our Any SUD group also showed higher baseline severity for BSL the before the intervention and significantly higher improvement on BSL. Possibly patients with more important symptoms show a faster short-term remission which may not be maintained over a longer period. This group of patients is often more stigmatized and perhaps the fact of them receiving special attention during therapy might motivate them to engage more. This could explain the greater symptom improvement. This however shoes that patients with comorbid SUD can benefit equally from treatment as patients without SUD. Further studies are needed to analyze these effects in short- and long-term outcomes.

There was no significant difference found in the delta OQ-45 measures for the two groups.

Again, this is an important finding as we hypothesized that the improvements would be less important in the Any SUD group. This result again shows that the intervention was also as effective independently from the presence of SUD.

Overall the therapeutic alliance progressed over the course of the therapy in all groups. In the group of patients with Any SUD the alliance increased more over time than in the other categories. This is an important finding and disproves our hypothesis.

This means that we cannot prove that SUD affects the therapeutic alliance. As before, a possible explanation could be the effect of TAU on SUD resulting in patients with SUD being able to focus on the therapy. It is also shown in other studies, that patients with SUD show high treatment alliance in individual therapy [55]. Again this shows that patients suffering from SUD can benefit from the GPM treatment and should not be excluded from treatment programs. As shown in other studies with cocaine dependent patients, drug use severity alone does not predict the alliance or time in treatment [37]. Strong alliance can predict better treatment retention with less drop-out and better drug outcome for some therapies (individual manual based drug counseling and brief psychodynamic therapy), in CBT high alliance is linked to shorter stay in treatment [55]. The ability to improve treatment retention with GPM may be a good preparation for long-term treatment if necessary.

The short-term approach to treatment can easily be applied to BPD patients with SUD by their health care practitioner to help diminish the intensity of central borderline problems and improve their functionality. If necessary at a later stage, these patients might be addressed for more in-depth treatment of BPD for which SUD is often a limiting factor.

There were a number of limitations for this study. We do not have any long-term outcome measures to prove that the effect of the treatment stays the same for both groups. These are secondary analysis, so we have fewer details on the evaluation of co-morbid SUD, such as severity of SUD. The number of patients for each group was limited. Furthermore, between substance use disorder analyses were not carried out due to the limited number of patients. Our outcome measures were self-reported which is subject to responder bias. Although the drop-out rate was moderate in our patient groups, it has to be considered as a possible limitation. It is a known that patients with SUD have a higher risk of treatment drop-out and perhaps complementary offers are needed to reduce this risk in order for patients being able to benefit even more from treatments such as GPM [56]. Another limitation for this study was the combination of two different treatment groups GPM and GPM + MOTR even though MOTR did not show a significant additional effect in previous studies.

## Conclusions

The short variant of GPM seems to be an effective treatment option for patients with BPD independently from the presence of SUD. Therefore, this treatment could be an effective entry-level treatment for patients with dual diagnosis and a possible preparatory step before starting more specific long-term treatment. It is important and possible to treat the BPD in patients with SUD. Treating the two disorders simultaneously could help enhance outcomes in both areas. Further studies are needed to confirm efficacy and long-term outcome.

## References

[1] Skodol AE, Gunderson JG, Pfohl B, Widiger TA, Livesley WJ, Siever LJ (2002). The borderline diagnosis I: psychopathology, comorbidity, and personaltity structure. Biol Psychiatry.

[2] Leichsenring F, Leibing E, Kruse J, New AS, Leweke F (2011). Borderline personality disorder. Lancet.

[3] Trull TJ, Sher KJ, Minks-Brown C, Durbin J, Burr R (2000). Borderline personality disorder and substance use disorders: a review and integration. Clin Psychol Rev.

[4] Brooner RK, King VL, Kidorf M, Schmidt CW, Bigelow GE (1997). Psychiatric and substance use comorbidity among treatment-seeking opioid abusers. Arch Gen Psychiatry.

[5] Weiss RD, Mirin SM, Griffin ML, Gunderson JG, Hufford C (1993). Personality disorders in cocaine dependence. Compr Psychiatry.

[6] Preuss UW, Johann M, Fehr C, Koller G, Wodarz N, Hesselbrock V (2009). Personality disorders in alcohol-dependent individuals: relationship with alcohol dependence severity. Eur Addict Res.

[7] Kienast T, Stoffers J, Bermpohl F, Lieb K (2014). Borderline personality disorder and comorbid addiction: epidemiology and treatment. Dtsch Arztebl Int.

[8] Barber JP, Frank A, Weiss RD, Blaine J, Siqueland L, Moras K (1996). Prevalence and correlates of personality disorder diagnoses among cocaine dependent outpatients. J Personal Disord.

[9] Langås A-M, Malt UF, Opjordsmoen S (2012). In-depth study of personality disorders in first-admission patients with substance use disorders. BMC psychiatry.

[10] Bowden-Jones O, Iqbal MZ, Tyrer P, Seivewright N, Cooper S, Judd A (2004). Prevalence of personality disorder in alcohol and drug services and associated comorbidity. Addiction.

[11] Darke S, Ross J, Williamson A, Teesson M (2005). The impact of borderline personality disorder on 12-month outcomes for the treatment of heroin dependence. Addiction.

[12] McMain S, Ellery M (2008). Screening and assessment of personality disorders in addiction treatment settings. Int J Ment Heal Addict.

[13] Stoffers-Winterling JM, Völlm BA, Rücker G, Timmer A, Huband N, Lieb K. Psychological therapies for people with borderline personality disorder. Cochrane Database Syst Rev. 2012, Issue 8. Art. No.: CD005652. doi:10.1002/14651858.CD005652.pub2. 10.1002/14651858.CD005652.pub2 PMC6481907 22895952

[14] Bender DS, Dolan RT, Skodol AE, Sanislow CA, Dyck IR, McGlashan TH (2001). Treatment utilization by patients with personality disorders. Am J Psychiatr.

[15] Barnicot K, Katsakou C, Bhatti N, Savill M, Fearns N, Priebe S (2012). Factors predicting the outcome of psychotherapy for borderline personality disorder: a systematic review. Clin Psychol Rev.

[16] Lee NK, Cameron J, Jenner L (2015). A systematic review of interventions for co-occurring substance use and borderline personality disorders. Drug and alcohol review.

[17] Gainey RR, Wells EA, Hawkins JD, Catalano RF (1993). Predicting treatment retention among cocaine users. Int J Addict.

[18] Stark MJ, Campbell BK (1988). Personality, drug use, and early attrition from substance abuse treatment. The American journal of drug and alcohol abuse.

[19] Carroll KM, Rounsaville BJ, Gawin FH (1991). A comparative trial of psychotherapies for ambulatory cocaine abusers: relapse prevention and interpersonal psychotherapy. The American journal of drug and alcohol abuse..

[20] Carroll KM, Rounsaville BJ, Gordon LT, Nich C, Jatlow P, Bisighini RM (1994). Psychotherapy and pharmacotherapy for ambulatory cocaine abusers. Arch Gen Psychiatry.

[21] Barber JP, Gallop R, Crits-Christoph P, Frank A, Thase ME, Weiss RD (2006). The role of therapist adherence, therapist competence, and alliance in predicting outcome of individual drug counseling: results from the National Institute Drug Abuse Collaborative Cocaine Treatment Study. Psychother Res.

[22] Laaksonen MA, Knekt P, Lindfors O (2013). Psychological predictors of the recovery from mood or anxiety disorder in short-term and long-term psychotherapy during a 3-year follow-up. Psychiatry Res.

[23] Ventegodt S, Thegler S, Andreasen T, Struve F, Enevoldsen L, Bassaine L (2007). Clinical holistic medicine (mindful, short-term psychodynamic psychotherapy complemented with bodywork) in the treatment of experienced physical illness and chronic pain. Sci World J.

[24] Gunderson JG (2014). Handbook of good psychiatric management for borderline personality disorder: American psychiatric pub.

[25] McMain SF, Guimond T, Streiner DL, Cardish RJ, Links PS (2012). Dialectical behavior therapy compared with general psychiatric management for borderline personality disorder: clinical outcomes and functioning over a 2-year follow-up. Am J Psychiatr.

[26] Bateman A, Fonagy P (2009). Randomized controlled trial of outpatient mentalization-based treatment versus structured clinical management for borderline personality disorder. Am J Psychiatr.

[27] Raven MC, Carrier ER, Lee J, Billings JC, Marr M, Gourevitch MN (2010). Substance use treatment barriers for patients with frequent hospital admissions. J Subst Abus Treat.

[28] Skule C, Berge T, Eilertsen E, Ulleberg P, Dallavara Lending H, Egeland J, et al. Levels of alcohol use and depression severity as predictors of missed therapy sessions in cognitive behavioural psycho-educational group treatment for depression. Addiction research & theory. 2016:1–6.

[29] Paris J. Stepped care: an alternative to routine extended treatment for patients with borderline personality disorder. Psychiatr Serv. 2013; 10.1176/appi.ps.201200451 23945913

[30] Chanen AM, Berk M, Thompson K. Integrating early intervention for borderline personality disorder and mood disorders. Harvard review of psychiatry. 2016; 10.1097/HRP.0000000000000105 27144298

[31] Choi-Kain LW, Albert EB, Gunderson JG (2016). Evidence-based treatments for borderline personality disorder: implementation, integration, and stepped care. Harvard Review of Psychiatry.

[32] Black D, Allen J, St John D, Pfohl B, McCormick B, Blum N (2009). Predictors of response to systems training for emotional predictability and problem solving (STEPPS) for borderline personality disorder: an exploratory study. Acta Psychiatr Scand.

[33] Gunderson JG (2016). The emergence of a generalist model to meet public health needs for patients with borderline personality disorder. Am J Psychiatr.

[34] Tomko RL, Trull TJ, Wood PK, Sher KJ (2014). Characteristics of borderline personality disorder in a community sample: comorbidity, treatment utilization, and general functioning. J Personal Disord.

[35] Van Asselt A, Dirksen CD, Arntz A, Severens JL (2007). The cost of borderline personality disorder: societal cost of illness in BPD-patients. European Psychiatry.

[36] Crits-Christoph P, Siqueland L, Blaine J, Frank A, Luborsky L, Onken LS (1999). Psychosocial treatments for cocaine dependence: National Institute on Drug Abuse collaborative cocaine treatment study. Arch Gen Psychiatry.

[37] Siqueland L, Crits-Christoph P, Gallop R, Barber JP, Griffin ML, Thase ME (2002). Retention in psychosocial treatment of cocaine dependence: predictors and impact on outcome. Am J Addict.

[38] Gunderson JG, Links P (2008). The borderline diagnosis. Borderline personality disorder A clinical guide.

[39] Kramer U, Berger T, Kolly S, Marquet P, Preisig M, de Roten Y (2011). Effects of motive-oriented therapeutic relationship in early-phase treatment of borderline personality disorder: a pilot study of a randomized trial. J Nerv Ment Dis.

[40] Kramer U, Kolly S, Berthoud L, Keller S, Preisig M, Caspar F (2014). Effects of motive-oriented therapeutic relationship in a ten-session general psychiatric treatment of borderline personality disorder: a randomized controlled trial. Psychother Psychosom.

[41] Caspar F. Plan analysis; in Eells TD (ed): Handbook of psychotherapy case formulation. New York: Guilford Press. 2007;2:221–89.

[42] Kolly S, Despland J-N, de Roten Y, Marquet P, Kramer U. Therapist adherence to good psychiatric practice in a short-term treatment for borderline personality disorder. J Nerv Ment Dis. 2016;204(7):489–93. 10.1097/NMD.0000000000000481 27187770

[43] First MB, Gibbon M (2004). The structured clinical interview for DSM-IV Axis I disorders (SCID-I) and the structured clinical interview for DSM-IV Axis II disorders (SCID-II).

[44] Lecrubier Y, Sheehan DV, Weiller E, Amorim P, Bonora I, Sheehan KH (1997). The MINI international neuropsychiatric interview (MINI). A short diagnostic structured interview: reliability and validity according to the CIDI. European psychiatry.

[45] Lambert M, Morton J, Hatfield D, Harmon C, Hamilton S, Reid R, et al. & Burlingame, GM (2004). Administration and scoring manual for the Outcome Questionnaire.45.

[46] Emond C, Savard K, Lalande G, Boisvert N, Boutin M, Simard V, editors. Propriétés psychométriques de la Mesure de l’Impact (MI-45), version francophone du Outcome Questionnaire-45 (OQ-45.2)[Psychometric characteristics of the OQ-45, French Version]. American College of Foot and Ankle Surgeons conference, Montreal, QC; 2004.

[47] Bohus M, Kleindienst N, Limberger MF, Stieglitz R-D, Domsalla M, Chapman AL (2008). The short version of the borderline symptom list (BSL-23): development and initial data on psychometric properties. Psychopathology.

[48] Horvath AO, Greenberg LS (1989). Development and validation of the working alliance inventory. J Couns Psychol.

[49] Corbière M, Bisson J, Lauzon S, Ricard N (2006). Factorial validation of a French short-form of the working alliance inventory. Int J Methods Psychiatr Res.

[50] Cohen J (1988). Statistical power analysis for the behavioral sciences.

[51] Laird NM, Ware JH. Random-effects models for longitudinal data. Biometrics. 1982:963–74. 7168798

[52] McCulloch CE, Neuhaus JM (2001). Generalized linear mixed models: Wiley online Library.

[53] Team RC (2013). R: a language and environment for statistical computing.

[54] Team RC. Package "nlme" CRAN. 2017; Retrieved from https://cran.r-project.org/web/packages/nlme/nlme.pdf. Accessed May 2017.

[55] Barber JP, Luborsky L, Gallop R, Crits-Christoph P, Frank A, Weiss RD (2001). Therapeutic alliance as a predictor of outcome and retention in the National Institute on Drug Abuse collaborative cocaine treatment study. J Consult Clin Psychol.

[56] Penzenstadler L, Machado A, Thorens G, Zullino D, Khazaal Y. Effect of case management interventions for patients with substance use disorders: a systematic review. Frontiers in psychiatry. 2017;8 10.3389/fpsyt.2017.00051 PMC5382199 28428761

